# A Scoping Review of Medical Mistrust Among Racial, Ethnic, and Gender Minorities With Breast and Ovarian Cancer

**DOI:** 10.7759/cureus.62410

**Published:** 2024-06-14

**Authors:** Manisha Antony, Emma Putnam, Cadynce Peltzer, Arkene Levy

**Affiliations:** 1 Medical Education, Nova Southeastern University Dr. Kiran C. Patel College of Osteopathic Medicine, Fort Lauderdale, USA; 2 Medical Education, Nova Southeastern University Dr. Kiran C. Patel College of Osteopathic Medicine, Clearwater, USA; 3 Medical Education (Pharmacology), Nova Southeastern University Dr. Kiran C. Patel College of Allopathic Medicine, Fort Lauderdale, USA

**Keywords:** cancer screening, breast cancer, ovarian cancer, health disparities, racial minority, medical mistrust

## Abstract

An overarching theme in clinical literature suggests an inherent mistrust among populations of color within the healthcare system and the importance of healthcare professionals to bridge this gap in care. This is especially true when addressing cancer care in underserved populations due to mistrust in providers, diagnostic tools, and treatments. Ovarian cancer is difficult to diagnose early in all populations; however, women of color who have an intrinsic mistrust of the medical community will delay or refuse screenings or treatments that could be greatly beneficial. Similarly, although breast cancer rates are high in women of color, many are reluctant to utilize genetic screenings or counseling services due to bad experiences with healthcare, both personally and within their community. Moreover, transgender patients are at a unique disadvantage, as they face barriers to accessing culturally competent care while also being at a higher risk for developing cancer. The objective of this study was to conduct a scoping review of the literature in order to synthesize knowledge about the climate of mistrust between medical providers and racial, ethnic, and gender minorities with breast cancer and ovarian cancer. It is imperative for healthcare workers to acknowledge medical mistrust and strive to reduce internalized bias, increase their availability to patients, and ensure patients feel heard, respected, and well cared for during visits. Improving care by physicians can enhance trust between underserved communities and healthcare workers, encouraging all people to actively seek proper medical care and cancer screening, potentially resulting in a reduction of mortality and morbidity rates.

## Introduction and background

Medical mistrust is a pertinent issue faced by marginalized communities in the United States due to ongoing social injustice and discrimination against those from underserved communities. Medical mistrust is the suspicion of the intentions and trustworthiness of the healthcare system by marginalized groups due to prior discrimination and mistreatment regarding their social, racial, and economic status [1]. This medical mistrust contributes to the poor quality of healthcare and treatment received by patients from underserved groups, leading to poor emotional and social well-being. There is a greater prevalence of medical mistrust in ethnic and racial minorities with cancer. For instance, there is greater mistrust and fatalism in Hispanic and African American patients with prostate cancer than in non-Hispanic Whites [2]. Some factors associated with increased medical mistrust include previous negative experiences and suspicions about the intentions of clinicians, insurance, education level, perceived discrimination, and reduced self-efficacy due to social stigmas [3].

The negative experiences and discrimination faced by racial and ethnic minorities impact not just the individual patient-provider interactions but also collectively impact and further marginalize this community of individuals with similar cultural backgrounds. African Americans as a community have faced many injustices contributing to the lack of trust they have in healthcare workers. One example is the story of Henrietta Lacks. She was an African American woman who went to Johns Hopkins Hospital in 1941 for a vaginal bleed, which was later diagnosed as cervical cancer [4]. Due to limited research on cancer at that time, the hospital obtained her cervical cells, now called HeLa cells, without informed consent while she was undergoing treatment. These cells were used for research purposes to study the behavior of cancer cells and led to the making of the first poliovirus vaccination [4]. Although HeLa cells have led to breakthroughs in medicine and are to this day used by researchers to make scientific discoveries on cancer, vaccinations, and viral illness, Henrietta Lacks’ cells and genomic data were obtained and utilized in research without her knowledge [4]. This case of Henrietta Lacks breaks several ethical principles in the field of medicine by infringing upon her autonomy. This underscores the reasons why many African American patients are hesitant to place their trust in healthcare providers, as they fear losing their freedom and ability to decide how their body and cells are utilized. Medical mistrust leads to lower utilization of genetic counseling and screening tools for various cancers [4]. Additionally, there is limited information and data on how this specifically impacts racial, ethnic, and gender minorities with breast and ovarian cancer. The objective of this study was to conduct a scoping review of the literature in order to synthesize knowledge about the climate of mistrust between medical providers and patients with breast cancer and ovarian cancer.

## Review

Methods

A literature review of medical mistrust among racial/ethnic and gender minorities with reproductive cancer was conducted using the PubMed database, Oxford Academic database, and Wiley Online Library. The search was conducted using the following search terms: “ovarian cancer,” “breast cancer,” “medical mistrust,” “racial minorities,” “genetic testing,” and “transgender.” The inclusion criteria for this literature review consisted of articles published after 2004, those within the United States population, and populations over the age of 18. The search was restricted to the population in the United States to avoid confounding variables stemming from cultural and healthcare system differences that exist between different countries. The initial search resulted in 15,667 articles, and of these, 63 articles were chosen to be screened. These 63 articles were then screened based on their titles and abstracts. If the titles or abstracts included research pertaining to ovarian or breast cancer and medical mistrust faced by Black, Hispanic, or transgender populations, they were analyzed. Thirty-one articles were excluded because they did not fit within the inclusion criteria listed above or did not contain information regarding medical mistrust or reproductive cancer. Thirty-two full-length articles were reviewed thoroughly, and all 32 articles were chosen for their current and applicable data analyses. This paper discusses mistrust among diverse populations with ovarian and breast cancer, including unique aspects related to transgender patients.

Ovarian cancer

Ovarian cancer prognosis is strongly associated with staging and grading at the time of diagnosis. Around 70% of epithelial ovarian cancer cases are present at stage III or stage IV, which correlates to an estimated 10-year survival of 23% and <8%, respectively [5,6]. Despite research and awareness efforts, it remains a challenge to make an early diagnosis, and ovarian cancer remains the most fatal gynecologic cancer [7]. This is partially due to the elusive symptoms associated with malignancy, which include feelings of abdominal distension, discomfort, and gastrointestinal symptoms [8]. Additionally, the absence of a recommended screening protocol due to a lack of sensitive and specific biomarkers for early-stage disease further contributes to late-stage diagnoses. Transvaginal sonography, genetic testing, and the cancer antigen 125 (CA 125) cancer marker are not exclusively specific to ovarian cancer, and a schematic is not available for the appropriate use of these tests [7]. While there are many active studies examining these biomedical barriers to encourage earlier diagnoses and more targeted therapies, it is important to also consider the social complication of medical mistrust, which further exacerbates the already challenging diagnostic landscape. Due to an inherent mistrust of the healthcare system, patients from minority groups with ovarian cancer can be unwilling to participate in trials or studies. Many randomized studies regarding cancer prevention in white populations with BRCA1 (BReast CAncer gene 1) and BRCA2 (BReast CAncer gene 2) mutation have been conducted, while similar studies are lacking in racial/ethnic and gender minority populations [9]. Due to these limited studies and long-term data on cancer prevention in minorities, ovarian cancer patients from minority groups are more hesitant to try genetic counseling as they are not aware of the complete risks and benefits [9]. It is important that practicing healthcare professionals are aware of these factors and take the time to establish trust with their patients by properly educating and alleviating any concerns the patients may have. One way to implement this is by assuring clinicians are adequately educated on genetic counseling, especially regarding racial and ethnic minorities, through Continuing Medical Education (CME). In the early 2000s, two community partners, QueensCare Health and Faith Partnership and Olive View-University of California, Los Angeles (UCLA) Medical Center, with a predominantly Hispanic population, were given five CME lectures regarding hereditary cancer knowledge prior to starting a cancer genetics service [10]. A survey administered before and after the CME lectures to evaluate the clinician knowledge found that there was a 66% to 94% increase in hereditary cancer knowledge. This contributed to the clinic’s improved genetic services as the number of referrals from the Hispanic population increased in a span of three years [10]. Similarly, broadening these CME seminars on genetic counseling among racial, ethnic, and gender minorities to clinics nationwide can help bridge the gaps in the knowledge of physicians, hence improving trust between physicians and ovarian cancer patients from marginalized minority communities and increasing the utilization of genetic services by these patients.

In addition to gaps in knowledge among physicians, there is limited genetic counseling and testing accessible to ovarian cancer patients from racial and ethnic minority communities, making it harder to get tested. One factor for this could be time limitation [9]. The genetic testing takes a minimum of one hour and is often only available during business hours. This makes it difficult for Black and Hispanic individuals, who are a part of the non-salary-based population, to take time off from work. These genetic services are also costly and found in suburban or urban areas, further reducing accessibility to under-resourced individuals due to travel expenses and time [9]. This also makes it difficult for family members to get tested, hence reducing the efficacy of genetic counseling as it is used to identify and treat hereditary ovarian cancer. Racial bias in the healthcare setting can also impact the lower rate of genetic service utilization by racial and ethnic minorities. A survey involving 100 students from genetic counseling programs in the United States and Canada reported an implicit racial bias in their program favoring the white population as these students had a lack of interaction with black healthcare professionals, a lack of effective diversity coursework, and a lack of exposure to diverse populations [11]. Around 38% of the participants also reported racial insensitivity by the supervising genetic counselors and physicians toward racial minorities [11]. This racial bias in healthcare, starting with training programs, contributes to the lack of trust among racial and ethnic minorities, making these individuals more reluctant to utilize genetic counseling. Additionally, these structural factors can increase medical mistrust as the medical resources are tailored toward a higher-income population that consists mostly of the white population [11]. This can be perceived by minorities as discrimination, making them unwilling to participate. Therefore, it is essential to have equitable opportunities in underserved areas, as well as develop more cost-efficient ways to perform genetic testing.

**Figure 1:**
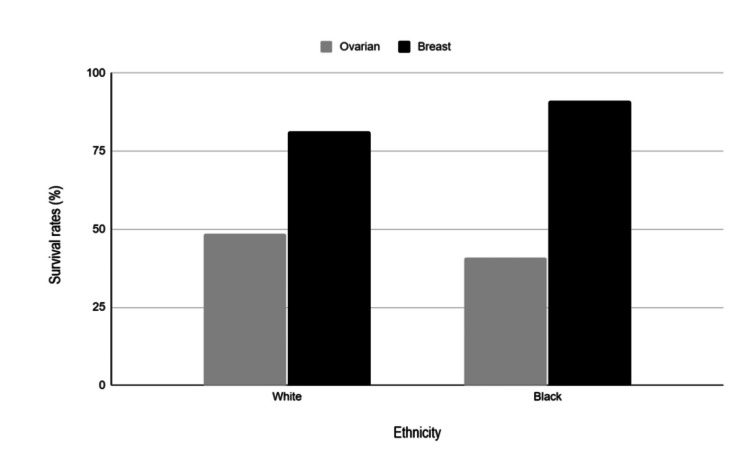
Report of cancer survival rates of females between 2005 and 2011 obtained from National Cancer Institute's Surveillance, Epidemiology, and End Results (SEER) incidence data. This is an original figure created by the authors using data from reference 13.

Black women with ovarian cancer are less likely to acknowledge the health benefits of genetic testing because they believe that results that reveal risk will contribute to further stigmatization and labeling as being “inferior” [12]. A key feature that contributes to this feeling of inferiority, which further perpetuates medical mistrust, is the fact that Black women with breast and ovarian cancer have lower survival rates compared to White women, as seen in Figure 1 [13]. Furthermore, the National Comprehensive Cancer Network provides thorough guidelines for ovarian cancer treatment that consist of cytoreductive or debulking surgery followed by chemotherapy, and a study done by the National Cancer Institute reported that black women received less treatment that followed these guidelines, contributing to the different survival rates of Black and White populations [12,14]. This demonstrates the inequality of treatment between whites and other racial groups, furthering the gap between healthcare and Black women as it causes mistrust.

Breast cancer

Breast cancer is the second leading cause of cancer deaths in women in the United States [15], and therefore, a timely diagnosis is critical to enable prompt treatment and improve prognosis. One diagnostic tool for breast cancer is the BRCA1/2 genetic testing and counseling. BRCA1/2 are two genes involved in homologous recombination repair [16]. Up to 10 % of hereditary breast cancer cases are usually associated with germline variants in BRCA1 and BRCA2 and more aggressive disease course. Furthermore, a germline mutation in the BRCA1 gene is associated with a higher lifetime risk (72%) for developing breast cancer than for BRCA2 mutation carriers (69%) [17]. Identifying these specific mutations can impact the treatment received by patients. Therefore, screening is important, especially for women who are at an increased risk of developing breast cancer. Some screening tools include annual mammograms and biopsies. However, these tools are most often effective once the cancer has already appeared.

There are disparities in the utilization of genetic testing by women of color. Black women have the highest rates of breast cancer recurrence and mortality compared to their white counterparts [13], yet Black women specifically are underutilizing genetic counseling and testing compared to white women [18]. Unfortunately, more than half of Black women surveyed in the Sheppard et al. study admitted to feelings of mistrust in the medical system [18]. The Sheppard et al. study looked at three groups of Black women to identify the sociocultural impact of genetic testing in this population [18]. The three groups contained women who were unaffected by breast or ovarian cancer, women who had a relative diagnosed with breast or ovarian cancer, and women who themselves had been diagnosed with breast or ovarian cancer [18]. The seven-point medical mistrust index (MMI) was used to determine a baseline of mistrust, followed by a few more surveys on self-efficacy as designed by the research team [18]. Many factors influence the level of mistrust in patients of color, including lack of availability of testing, discrimination, and lack of patient education [19]. A study done by Haken et al. explores how a diverse healthcare team leads to increased care for minority populations [20]. This paper shows the need for more diversity in healthcare because providers of minority populations know the biases and concerns in their communities [20]. Therefore, they are in a position to practice in a way that will help to decrease these inequalities. [20] Increasing diversity in this field can help build trust and improve the utilization of testing [20,21].

Just under half of the participants in the Sheppard et al. studies were knowledgeable about genetic counseling and testing services for breast cancer that were available to them [18]. Of that percentage, only 30% followed up and utilized these services [18]. Historically, minority communities have not had success with traditional research techniques, partnered with an institutionalized mistrust of the system that leads them to be less likely and willing to try new therapies. This is to protect themselves from being taken advantage of by the system, as many of their families have been in the past. In a survey of Black Bostonians, it was found that while some had generally positive feelings about clinical trials, the majority associated them with feelings of fear and exploitation [22]. One reason for this is the Tuskegee syphilis study from 1932 to 1972, in which medical experiments were conducted without informing or gaining consent from the participants who were African Americans and exposing them to syphilis [23]. This makes patients of various ethnicities question the ethics and morals of genetic counselors due to predisposed discrimination and unfair treatment.

Transgender patients

The term “transgender” describes persons whose gender identity or gender expression does not conform to that typically associated with the sex they were assigned at birth. Some transgender patients undergo hormonal treatment with the aim to feminize or masculinize their appearance and can develop a wide variety of cancers, including breast and ovarian [24].

Transgender patients who develop cancer often get diagnosed at a later stage due to inequity in access to cancer screening facilities [23]. Many transgender patients have previously experienced discrimination or mistreatment in healthcare settings, which deters them from seeking out screening opportunities [25]. Often, they have not received care that is appropriate and sensitive to their specific needs due to instances of misgendering or lack of provider education.

Studies show that gender-affirming hormone therapy and transgender mastectomy surgery increase the risk of breast cancer in transmasculine patients [26,27]. For instance, an analysis of medical records of 318 patients who went through chest reconstructive surgery found that 6.6% of the transmasculine patients had an increased risk of breast cancer, with 1.2% having greater than two times the risk [26]. Similarly, data from the Veteran Health Administration reported seven cases of breast cancer in transmasculine patients, with 52% having received gender-affirming hormone therapy [27]. The incidence of breast cancer is much lower in transgender patients compared to cisgender women; however, clinicians should take caution and enforce cancer screening in transgender patients due to the increased risk of breast cancer. In addition, there is no current evidence of increased risk for ovarian cancer among transgender patients compared to cisgender patients, so ovarian cancer screening among these patients is not recommended [27].

Furthermore, following a reduction mammoplasty surgery, patients are still left with some breast tissue, which increases the risk for breast cancer. However, since there is a massive change in breast contour, mammography is not as effective in detecting malignant changes [25]. In these cases, genetic counseling plays a larger role. However, transgender patients are reluctant to access cancer screening facilities because of medical mistrust resulting from previous experiences of poor quality of care, stigmatization, discrimination, and lack of openness with their healthcare providers [23]. Studies show that limited knowledge of physicians and other providers about transgender patients contributes to poor quality of care as patients do not get adequate history taking and standard physical exams. An anonymous cross-sectional phone survey was conducted with physicians and other providers from nine Obstetrics and Gynecology Departments across the United States, and they were asked a series of questions regarding the care of transgender patients [27]. When asked about transgender male-to-female patients, only 35.3% of providers were comfortable with providing them with care, 80.4% of providers were willing to perform routine breast examinations, and 59.4% did not know the recommendations for breast cancer screening in these patients. When asked about transgender female-to-male patients, only 29% of the providers were comfortable caring for them, and 88.7% were willing to perform pap smears on the patients [27].

This study highlights the importance of providing appropriate education for physicians to improve their knowledge and cultural competence in caring for transgender patients. This education, including relevant standard physical exams, history taking, communication, and appropriate use of pronouns, has been shown to improve the quality of care, decrease institutional discrimination, and encourage transgender patients to be more open with providers [27,19]. Moreover, it is essential to develop infrastructure that can incorporate changes for routine exams required for transgender patients, as this increases access to care. Addressing the above factors can mitigate and reduce medical mistrust and enable transgender patients to access modalities like genetic testing that can reduce mortality rates from cancer [27,19].

The relationship between health literacy, patient education, and medical mistrust

It is well documented that adequate patient education is very important for increasing trust between healthcare providers and patients. Providing education is widely known to help patients manage their condition and navigate the health care system [28]. It has been found that patients who report trust in their primary care and oncology providers have higher levels of adherence to cancer screenings and report higher amounts of satisfaction with their care [29]. This is extremely important because earlier screenings and increased screening adherence lead to earlier diagnoses and better outcomes [29]. Adequate patient education is correlated with decreased emergency department visits and re-admissions to the hospital [28]. This would be important for patients who are an underserved population and cannot afford multiple hospital visits. One challenge that has been identified with providing education to patients is that it requires multiple doctor visits, which can be difficult for many reasons [28]. For healthcare providers to be able to individualize educational programs for their patients and to ensure they make well-informed decisions about their health, they must be aware of factors that affect the patient’s situation [27]. In addition to awareness of factors that contribute to medical mistrust, providers should actively search for and attend training to improve their communication skills with underserved populations [30]. The study completed by Hall et al. concludes that the more recent training an oncologist had attended, the more they were able to discuss relevant concerns and challenges to patients of African American descent [30].

**Figure 2:**
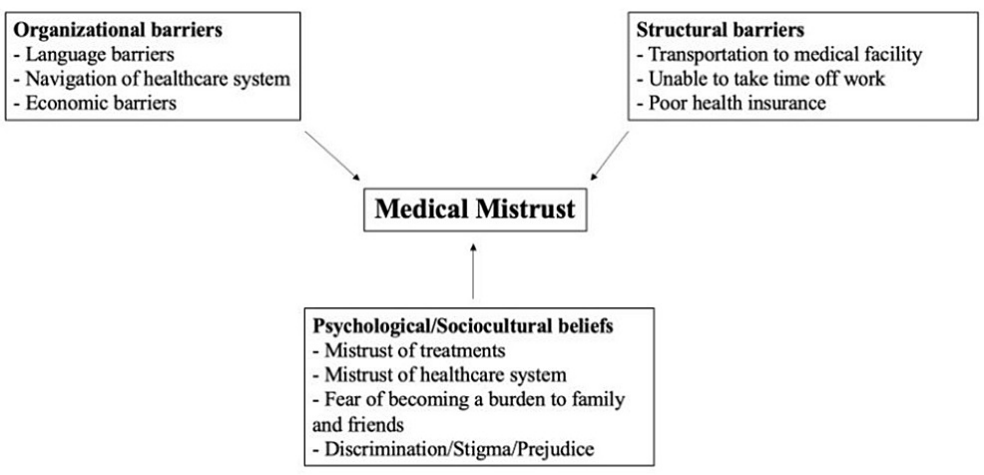
Summarization of contributory factors to why patients who belong to minority groups have inherent medical mistrust. This is an original figure created by the authors.

Patients may be struggling with structural, organizational, psychological, sociocultural factors or a combination of each category, as seen in Figure 2 [9,19,28]. Patients may face structural difficulties such as living in an area where they do not have access to a clinic, whether it be because they cannot arrange transportation or they cannot afford to go to clinics near them [31]. They also may not be able to take time off of work or arrange for childcare to go to an appointment [9]. Their difficulties may be more organizational, and they may have trouble navigating the healthcare system [32]. It is often unfamiliar and confusing, and adding a language barrier on top of that may make it seem impossible to attain care, as they may be concerned about insurance or the costs of the visit [32].

It is important to be cognizant of these factors because, for these patients, there is an institutionalized, inherent barrier to care. We must address these root concerns in order to increase accessibility to these screening tools and to dispel any mistrust or fear surrounding diagnostic tests and novel therapies that may be able to detect and treat early stages of cancer. Moreover, there are limited studies and research found on genetic testing involving minorities because they are unwilling to participate [19]. If the gap between healthcare and minorities is bridged, they will be more open to participating in trials, hence opening up more opportunities for advancing research.

## Conclusions

It is well established in the literature that there is an inherent mistrust in populations of color for the medical system, making them reluctant to follow up with diagnostic tools and treatments, especially regarding cancer screenings and genetic counseling. The structural challenges faced by racial, ethnic, and gender minorities, such as fear of stigma, inability to take time off work for appointments, or negative healthcare experiences, increase mistrust in the community. It is imperative for healthcare workers to acknowledge this, strive to educate themselves to bridge cultural gaps, and increase their availability to patients to ensure they feel safe and well cared for during visits.
